# Meta-emotional intelligence in cochlear-implanted preadolescents and adolescents

**DOI:** 10.1007/s00405-025-09626-z

**Published:** 2025-09-18

**Authors:** Antonella D’Amico, Maria Nicastri, Domenico Cuda, Letizia Guerzoni, Patrizia Mancini, Alessandro Geraci

**Affiliations:** 1 Department of Psychology, Educational Science and Human Movement, University of Palermo, Viale delle Scienze, Edificio 15, Palermo, 90128 Italy; 2 Department of Sense Organs, University Sapienza, Rome, Italy; 3 Department of Medicine and Surgery, University of Parma, Parma, Italy; 4 Department of Otorhinolaryngology, “Guglielmo da Saliceto” Hospital, Piacenza, Italy

**Keywords:** Cochlear implants, Emotional abilities, Meta-emotional intelligence, Adolescents, Preadolescents

## Abstract

**Purpose:**

This study investigates differences in meta-emotional beliefs and meta-emotional intelligence between preadolescents and adolescents with cochlear implants (CIs) and their typically hearing (TH) peers.

**Methods:**

The sample included 86 participants aged 10–18 years, evenly divided between those with CIs and TH individuals. The IE-ACCME test, a multi-method tool, was used to assess meta-emotional intelligence. Statistical analyses were carried out to compare meta-emotional intelligence dimensions between CIs and TH groups.

**Results:**

The findings reveal that the CIs group had significantly higher overall meta-emotional belief scores, suggesting that individuals with CIs perceive emotions as playing a more significant role in their lives. Despite this, no significant differences were found in subscales related to emotion perception, comprehension, and management, indicating similar beliefs across both groups. However, the CIs group scored higher on the facilitation subscale, reflecting stronger beliefs about using emotions to enhance thinking. Additionally, the CIs group tended to overestimate their emotional abilities, both in everyday life and in emotional ability tests.

**Conclusion:**

CIs group exhibited heightened meta-emotional beliefs and a tendency to overestimate their emotional abilities, reflecting a distinct meta-emotional intelligence profile linked to hearing loss and cochlear implantation. These findings suggest a distinct emotional profile for individuals with CIs, highlighting the need for targeted emotional and meta-emotional skills training.

## Introduction

Hearing impairments during childhood can significantly impact various facets of cognitive development, such as language acquisition, verbal communication, reading proficiency, academic achievement, and emotional development [1]. In terms of emotional development, research has shown that children with hearing impairments are more likely to experience internalizing difficulties, such as anxiety and depression, as well as externalizing behaviors like hyperactivity and conduct issues, compared to their typically hearing (TH) peers [2, 3]. A study [4] explored psychosocial development in children using hearing aids (HA) or cochlear implants (CI), finding that many children with hearing impairments face an elevated risk of psychopathology and social developmental challenges compared to the TH population. Factors such as the absence of additional disabilities, higher non-verbal cognitive ability, language proficiency, and functional communication skills were identified as significant predictors of improved psychosocial functioning.

The intricate relationship between language and psychosocial development is well-established [5], particularly in deaf and hard-of-hearing (DHH) populations. Challenges in verbal communication, speech production, and language comprehension are often cited as contributing factors to social difficulties in these individuals [6, 7]. DHH children often experience delays in language development, particularly in acquiring spoken and auditory language. However, it is not solely hearing loss or language ability but rather communication competence that appears to be pivotal for social interactions in DHH children [8]. Indeed, DHH children often exhibit differences in interaction and communication, such as turn-taking, joint attention, and interpreting nonverbal cues [9, 10]. These difficulties can lead to greater impulsivity and challenges in regulating emotions effectively. Additionally, their vocabulary for expressing emotions may be limited due to their language deficits. Without access to linguistic symbols, such as spoken or signed language, deaf children may struggle to internally process and understand their emotional experiences. Consequently, these challenges can result in significant gaps in their socio-emotional development, affecting their ability to form relationships, communicate, and regulate their behavior effectively [11].

Building on these observations, it was recently conducted a study to explore emotional intelligence in individuals with long-term cochlear implants (CIs), aiming to understand if early implantation (ages 7–48 months) may protect children from developing emotional problems [12]. Emotional intelligence was examined using the four-branch ability EI model [13, 14] and the Italian IE-ACCME test [15]. The study [12] compared emotional abilities in perception, cognitive facilitation, understanding, and emotion management between CI users and age- and gender-matched TH controls. The findings revealed no significant differences in emotional perception of faces and images or cognitive facilitation and interpersonal emotion management. However, the CI group showed difficulties in understanding emotional blends but excelled in managing personal emotions. Additionally, gender differences within the CI group showed males outperforming females in the cognitive facilitation of sensations. Several correlations between emotional abilities and factors such as age at CI, nonverbal IQ, and lexical skills were identified. These results suggest that cochlear implantation can positively influence emotional development, enabling children with CIs to achieve age-appropriate emotional skills in perception and cognitive facilitation and surpass expectations in personal emotion management. However, the study also indicated that cochlear implantation does not entirely prevent emotional difficulties, particularly in areas related to language development, such as emotional understanding. For these reasons, results emphasize the importance of EA assessments in CI users to monitor developmental trajectories and identify potential emotional disorders early, thus enabling timely interventions.

Building on these findings, the current study aims to further explore the nuances of emotional development in adolescents with CIs, with a particular focus on Meta-Emotional Intelligence (MEI), which involves awareness and regulation of one’s emotional processes. MEI is a multidimensional construct based on both the ability-EI model [13, 14] and metacognition theories [16]. Meta-emotional intelligence arises from the combination of emotional abilities and meta-emotional dimensions, such as beliefs about emotions, self-concept regarding one’s emotional abilities, and self-evaluation of performance in emotional tasks [17].

Previous studies [18–20] have demonstrated that individuals’ levels of emotional abilities measured in performance tests and self-perceived emotional abilities measured using self-report scales do not always correspond and are often not correlated. This discrepancy occurs because measures of ability EI and self-reported EI evaluate distinct psychological processes within the same construct, as self-perception is not always accurate, and believing one possesses certain abilities does not necessarily mean they can use them effectively [19]. This may also result from low meta-emotional knowledge, as people are not always aware of their emotional abilities, tending to underestimate or overestimate them in self-report assessments [17]. In a study investigating age and sex differences in MEI [21], findings revealed that in the preadolescent group, both boys and girls slightly overestimated their emotional competencies. However, as they transitioned into adolescence, boys increasingly overestimated their everyday emotional abilities, while girls tended to underestimate theirs. This contrasting trend extended to meta-emotional self-evaluation, with boys showing a greater inclination to overestimate their performance in ability tests compared to girls, who were more inclined toward underestimation. Additionally, girls exhibited higher levels of meta-emotional beliefs compared to boys across both age groups. In another study [22], findings revealed that individuals with sufficient meta-emotional knowledge are more socially accepted by their peers compared to those who overestimate their abilities and consequently face more rejection. Similarly, adolescents with accurate meta-emotional self-evaluation experience greater acceptance and lower rates of peer rejection. Additionally, a positive association was found between preadolescents’ psychological well-being and their meta-emotional beliefs. These results demonstrated that MEI is critical for adaptive functioning and social competence.

Thus, by extending the scope of previous research [12] to include MEI, this study seeks to investigate how adolescents with CIs perceive, understand, and manage their emotions at a meta-level, thereby contributing to a more detailed understanding of the long-term outcomes of cochlear implantation on emotional and social competencies.

## Materials and methods

### Participants

The study involved an overall sample of 86 preadolescents and adolescents (36 males and 50 females) ranging from 10 to 18 years (average age = 14.46, SD = 2.40). The sample included 43 preadolescents and adolescents with cochlear implants (CIs) and the other 43 of typically hearing (TH) young people. The group with cochlear implants (CIs), consisted of 25 females and 18 males, aged between 10.9 and 18 years, with an average age of 14.4 years (SD = 2.42). All participants were diagnosed with congenital profound hearing loss (90–110 dB HL) by the age of three, averaging 13.3 months at diagnosis (SD = 9.6, range 3–36 months). The first CI was implanted at an average age of 22.6 months (SD = 11.02, range 7–48 months). Most participants utilized bilateral devices (21 with bilateral CIs and 2 with bimodal configurations). They all had full electrode array insertions and no anatomical or functional cochlear or acoustic nerve abnormalities. Non-verbal IQs, evaluated using the Raven Progressive Matrices [23], were normal, scoring above the 25th percentile (average = 73.09 percentile, SD = 17.48). The duration of CI use was between 7 and 15.75 years, averaging 12.51 years (SD = 2.25). CI Sound Free Field threshold was 25.61 dB HL (SD = 4.23, range 19–34 dB HL). Speech perception scores, assessed by the Matrix Sentence Test [24], ranged from − 6.01 to 6.2 dB (mean = −2.01 dB, SD = 2.41). Language abilities, measured through the verbal denomination tasks from the BVN 5–11 [25] and BVN 12–18 [26], resulted in a mean z-score of −0.45 (SD = 1.24, range − 4.7 to 1.2), with 30.2% (13 participants) scoring below the standard distribution. The group of typically hearing (TH), defined by a PTA of ≤ 10 dB HL (mean = 3.79 dB, SD = 3.24, range 0–10 dB), consisted of 18 males and 25 female ranging from 10 to 18 years (average age = 14.53, SD = 2.40). Non-verbal IQs were normal scoring above the 25th percentile (average = 72.4 percentile, SD = 17.39).

### Instruments

MEI was evaluated using the IE-ACCME test [15], an Italian multi-method assessment tool tailored for preadolescents and adolescents. The test comprises four components: (1) a questionnaire on meta-emotional beliefs (CE); (2) a self-report scale on emotional self-concept (CME); (3) a performance-based emotional abilities test (AE); and (4) a self-rating scale for evaluating one’s performance on the AE test (AP).

Meta-Emotional Beliefs (CE) is a 16-item questionnaire that assesses beliefs about the role of emotions in everyday life, including perception, sensation, facilitation of thought, and the understanding and regulation of emotions. Responses are rated on a five-point Likert scale from “not true” (0) to “definitely true” (4).

Emotional Self-Concept (CME) is a 20-item scale explores individuals’ perceptions of their emotional skills. Participants self-evaluate their ability to recognize emotions in faces, images, and feelings, use emotions in thought processes, understand emotional vocabulary and transformations, and manage emotional states personally and interpersonally, using a scale from 0 to 4 (“not true” to “definitely true”).

Emotional Abilities Test (AE) is a performance-based test consisting of 60 items across eight tasks, measuring emotional abilities in perception (faces and pictures), facilitation (use and sensations), understanding (blends and transformations), and management (personal and interpersonal). The AE scale is based on the EI ability model [13, 14] and it employs a general and expert consensus methodology, with scores based on the frequency of selected responses in standardization samples [27]. The general sample included 1,084 Italian preadolescents and adolescents, while the expert sample comprised 40 Italian emotion scholars.

Self-Rating of Performance (AP) is an 8-item scale embedded within the emotional abilities test, where respondents self-evaluate their performance after each task using a six-point Likert scale from “not good at all” (0) to “very good” (5).

All scores for the CE, CME, AE, and AP components are standardized, with a mean of 100 and a standard deviation of 15. This standardization facilitates comparison across the different scales, enabling the computation of meta-emotional knowledge and meta-emotional self-evaluation scores.

Meta-Emotional Knowledge score (CMeta) represents the discrepancy between the emotional ability test (AE) scores and the emotional self-concept scale (CME) scores. It indicates how well an individual’s performance on the ability test aligns with their perceived emotional abilities in everyday life. A discrepancy greater than 15 standardized points suggests low meta-emotional knowledge. Discrepancies can indicate overestimation (positive scores) or underestimation (negative scores) and are categorized as low (below ± 15), medium (between ± 15 and ± 30), and high (between ± 30 and ± 45).

Meta-Emotional Self-Evaluation score (AvMeta) measures the discrepancy between the emotional ability test (AE) scores and the self-rating about performance scale (AP) scores. It reflects the extent to which an individual’s performance on the ability test matches their self-assessment of their performance. A discrepancy greater than 15 standardized points indicates low meta-emotional self-evaluation. Discrepancies can also indicate overestimation (positive scores) or underestimation (negative scores) and are categorized as low (below ± 15), medium (between ± 15 and ± 30), and high (between ± 30 and ± 45). Both meta-emotional knowledge and meta-emotional self-evaluation scores can have positive or negative values. Positive values indicate an overestimation of one’s emotional abilities in daily life and/or testing situations, whereas negative values indicate an underestimation of one’s emotional abilities in these contexts.

#### Data analysis

All statistical analyses were conducted using Statistical Package for Social Sciences 25.0 [28]. Means and standard deviations were computed for all variables of interest. The Shapiro-Wilk test was employed to examine the normality of data distribution and determine the appropriate statistical approach (parametric vs. nonparametric). For normally distributed data, a t-test was utilized to evaluate statistical differences between the CI/TH groups. For non-normally distributed data, the nonparametric Mann-Whitney U test was applied. In instances where data indicated a normal distribution according to the Shapiro-Wilk test, but the Levene test revealed significant differences in variance, the one-way ANOVA with Welch-Satterthwaite correction was employed to adjust the degrees of freedom [29].

## Results

### Demographic, audiological, and implant characteristics

Details of the demographic and audiological data are presented in Table 1, while the characteristics of the cochlear implants are provided in Table 2. Table 3 presents the descriptive statistics and statistical tests for group differences (CI and TH) in CE scale, CMeta, and AvMeta scores. For non-parametric tests such as the Mann-Whitney U test, median standard scores (Mdn) and interquartile ranges (IQR) are reported. For parametric tests, mean standard scores (M), standard deviations (SD), and mean differences are reported.

**Table 1:** Demographic characteristics and audiological data of the study and control groups

Categorical variables		CI	TH	
Gender	female male	25	25	
		18	18	
Economic Income	low	1	2	
	medium-low	4	3	
	medium	28	28	
	medium-high	10	10	
	high	0	0	
Continuous Variables		Mean (SD)	Mean (SD)	p-values
Age at assessment (yrs)		14.4 (2.42)	14.53 (2.39)	0.77
Maternal education Level (yrs of study)		15.16 (2.92)	15.33 (2.96)	0.76
Non Verbal IQ (percentile)		73.09 (17.48)	72.4 (17.39)	0.82
Age at diagnosis (mths)		13.3 (9.6)	/	
Age at cochlear implant (mths)		22.67 (11.02)	/	
CI use (yrs)		12.51 (2.25)	/	
Pre CI PTA (dB HL)		104.64 (9.75)	/	
Post CI Free Field threshold (dB HL)		25.61 (4.23)	/	
Matrix Sentence Test (SRT)		−2.01 (2.41)	/

**Table 2:** Details of cochlear implants characteristics

CI		*n*
Listening Mode	Unilateral CI	20
	Bilateral CI	21
	Bimodal	2
Cochlear implants	Cochlear^®^	20
	AB^®^	17
	Med-El	6
Strategies	ACE™	20
	Hi-Res120™	17
	FS4	6

**Table 3:** Descriptive statistics and independent sample tests for group differences (CI and TH) in CE, CMeta, and AvMeta

Scale	Group	N	M/Mdn	SD/IQR	Mean difference	Statistic	*p*-value	Effect size
CE-TOT^a^	TH	43	81.58	12.48	–21.46	Welch’s t = − 6.31; df = 73	< 0.001	Cohen’s d = − 1.36
	CI	43	103.05	18.49				
CE-P	TH	43	103.18	27.23		Mann-Whitney U = 892	0.781	Rank-biserial *r* = 0.04
	CI	43	103	18				
CE-F	TH	43	66.57	14.49		Mann-Whitney U = 120	< 0.001	Rank-biserial *r* = 0.87
	CI	43	99	25				
CE-C	TH	43	106.06	22.31		Mann-Whitney U = 906	0.876	Rank-biserial *r* = 0.02
	CI	43	106	22				
CE-G^a^	TH	43	104.48	17.84	–4.34	Student’s t = − 1.28; df = 84	0.326	Cohen’s d = − 0.28
	CI	43	108.81	13.26				
CMeta-TOT	TH	43	−0.00	0.39		Mann-Whitney U = 663	0.024	Rank-biserial *r* = 0.28
	CI	43	0.09	0.24				
CMeta-P	TH	43	0.03	0.38		Mann-Whitney U = 747	0.126	Rank-biserial *r* = 0.19
	CI	43	0.08	0.35				
CMeta-F	TH	43	0.10	0.20		Mann-Whitney U = 747	0.126	Rank-biserial *r* = 0.19
	CI	43	0.19	0.19				
CMeta-C	TH	43	−0.02	0.41		Mann-Whitney U = 741	0.114	Rank-biserial *r* = 0.20
	CI	43	0.07	0.30				
CMeta-G	TH	43	−0.06	0.23		Mann-Whitney U = 829	0.412	Rank-biserial *r* = 0.10
	CI	43	−0.01	0.26				
AvMeta-TOT	TH	43	−0.03	0.36		Mann-Whitney U = 669	0.028	Rank-biserial *r* = 0.28
	CI	43	0.09	0.36				
AvMeta-P	TH	43	−0.00	0.33		Mann-Whitney U = 786	0.233	Rank-biserial *r* = 0.15
	CI	43	0.11	0.26				
AvMeta-F	TH	43	0.08	0.32		Mann-Whitney U = 810	0.325	Rank-biserial *r* = 0.12
	CI	43	0.15	0.32				
AvMeta-C	TH	43	−0.14	0.40		Mann-Whitney U = 568	0.002	Rank-biserial *r* = 0.39
	CI	43	0.08	0.30				
AvMeta-G	TH	43	−0.00	0.26		Mann-Whitney U = 783	0.223	Rank-biserial *r* = 0.15
	CI	43	0.06	0.23				

*IQR* Interquartile range, *M* Mean, *Mdn* Median, *SD* Standard deviation, *TH* Typical hearing, *CI* Cochlear implant.

^a^ For CE-TOT and CE-G mean scores and standard deviation values are reported.

### Differences in meta-emotional beliefs

The TH group had a significantly lower mean CE-TOT score (M = 81.58, SD = 12.48) compared to the CI group (M = 103.05, SD = 18.49). The mean difference of − 21.46 was statistically significant (Welch’s t_(73)_ = − 6.31, *p* < 0.001) with a large effect size (Cohen’s d = − 1.36). This suggests a substantial difference in total CE scores between the groups, with the CI group presenting higher scores.

No significant difference was found in CE-P scores (U = 892, *p* = 0.781) between the TH group (Mdn = 103.18, IQR = 27.23) and the CI group (Mdn = 103, IQR = 18), with a negligible effect size (rank-biserial *r* = 0.04). This indicates that the scores on CE-P were similar for both groups.

The TH group scored significantly lower (Mdn = 66.57, IQR = 14.49) than the CI group (Mdn = 99, IQR = 25) on the CE-F subscale. The difference was highly significant (U = 120, *p* < 0.001), with a large effect size (rank-biserial *r* = 0.87). This substantial effect size indicates a profound disparity in CE-F scores between the groups.

There was no significant difference in CE-C scores (U = 911, *p* = 0.910) between the TH group (Mdn = 106.06, IQR = 22.31) and the CI group (Mdn = 106, IQR = 22), with a negligible effect size (rank-biserial *r* = 0.02). This suggests that the CE-C scores are comparable across both groups.

The TH group (M = 104.48, SD = 17.84) and the CI group (M = 108.81, SD = 13.26) did not differ significantly on CE-G scores (t_(84)_ = − 1.28, *p* = 0.326), with a small effect size (Cohen’s d = − 0.28). This indicates that there is no meaningful difference in general CE scores between the groups.

### Differences in meta-emotional scores

The mean CMeta-TOT score for the TH group (Mdn = −0.00, IQR = 0.39 was significantly lower than that of the CI group (Mdn = 0.09, IQR = 0.24), with a small to medium effect size (rank-biserial *r* = 0.28). This suggests a modest yet statistically significant difference in overall CMeta scores (U = 663, *p* = 0.024), with the CI group presenting a tendency to overestimate their emotional abilities in everyday life compared to the TH group.

No significant difference was found in CMeta-P scores (U = 747, *p* = 0.126) between the TH group (Mdn = 0.03, IQR = 0.38) and the CI group (Mdn = 0.08, IQR = 0.35), with a small effect size (rank-biserial *r* = 0.19). The TH group (Mdn = 0.10, IQR = 0.20) and the CI group (Mdn = 0.19, IQR = 0.19) did not differ significantly on CMeta-F scores (U = 747, *p* = 0.126), with a small effect size (rank-biserial *r* = 0.19). No significant difference was found in CMeta-C scores (U = 741, *p* = 0.114) between the TH group (Mdn = −0.02, IQR = 0.41) and the CI group (Mdn = 0.07, IQR = 0.30), with a small effect size (rank-biserial *r* = 0.20). The CMeta-G scores were not significantly different (U = 829, *p* = 0.412) between the TH group (Mdn = −0.06, IQR = 0.23) and the CI group (Mdn = −0.01, IQR = 0.26), with a small effect size (rank-biserial *r* = 0.10).

Regarding the AvMeta scores, the TH group (Mdn = −0.03, IQR = 0.36) scored significantly lower than the CI group (Mdn = 0.09, IQR = 0.36) on AvMeta-TOT (U = 669, *p* = 0.028), with a small to medium effect size (rank-biserial *r* = 0.28). This indicates a modest but statistically significant difference in overall AvMeta scores, as the CI group individuals showed a tendency to overestimate their performance in the ability test.

No significant difference was found in AvMeta-P scores (U = 786, *p* = 0.233) between the TH group (Mdn = −0.00, IQR = 0.33) and the CI group (Mdn = 0.11, IQR = 0.26), with a small effect size (rank-biserial *r* = 0.15). The TH group (Mdn = 0.08, IQR = 0.32) and the CI group (Mdn = 0.15, IQR = 0.32) did not differ significantly on AvMeta-F scores (U = 810, *p* = 0.325), with a small effect size (rank-biserial *r* = 0.12).

The AvMeta-C scores were significantly lower (U = 568, *p* = 0.002) in the TH group (Mdn = −0.14, IQR = 0.40) compared to the CI group (Mdn = 0.08, IQR = 0.30), with a medium effect size (rank-biserial *r* = 0.39). This demonstrates a notable difference in AvMeta-C scores, with the CI group showing an overestimation of the performance in the ability test.

Lastly, there was no significant difference in AvMeta-G scores (U = 783, *p* = 0.223) between the TH group (Mdn = −0.00, IQR = 0.26) and the CI group (Mdn = 0.06, IQR = 0.23), with a small effect size (rank-biserial *r* = 0.15). This suggests that AvMeta-G scores are relatively similar between the groups.

### Sex Differences in meta-emotional beliefs and metaemotional scores

The data were tested for sex differences between the TH and CI groups for the MetaEmotional Beliefs Scale and MetaEmotional Scores. Results show no sex differences within the TH group, whereas for the CI group, significant sex differences were found in CE-F (Mdn_M_ = 91, IQR_M_ = 30; Mdn_F_ = 110, IQR_F_ = 0.42; U = 128, *p* = 0.016, Rank-biserial *r* = 0.43) and AvMeta-F (Mdn_M_ = 0.02, IQR_M_ = 0.31; Mdn_F_ = 0.16, IQR_F_ = 0.30; U = 143, *p* = 0.043, Rank-biserial *r* = 0.36). These results indicate that girls of the CI group present higher metaemotional beliefs about using emotion in everyday life, but they also overestimated their facilitation of emotional abilities in the ability test.

## Discussion

The present study aimed to investigate differences in meta-emotional beliefs and meta-emotional intelligence between preadolescents and adolescents with cochlear implants (CIs) and their typically hearing (TH) peers. The findings highlight several key differences, providing insights into the unique emotional and cognitive profiles associated with hearing loss and cochlear implantation.

The results indicate that the CI group had significantly higher overall meta-emotional belief scores (CE-TOT) compared to the TH group. This suggests that individuals with CIs believe emotions play a more significant role in their everyday lives, which may reflect their unique social and communicative experiences. Interestingly, no significant differences were found in the meta-emotional beliefs about emotion perception (CE-P), comprehension (CE-C), and management subscales (CE-G), suggesting that both groups share similar beliefs. However, the CI group scored significantly higher on the facilitation subscale (CE-F), which assesses meta-emotional beliefs about the use of emotions to facilitate thinking.

The study also found significant differences in meta-emotional knowledge (CMeta-TOT) and self-evaluation (AvMeta-TOT) scores. The CI group demonstrated a tendency to overestimate their emotional abilities both in everyday life and in the emotional ability test compared to the TH group. This overestimation may reflect a compensatory mechanism or higher self-confidence in emotional abilities due to the unique social challenges and adaptations experienced by CI users. Specific subscale analyses revealed no significant differences in the perception (CMeta-P) and facilitation (CMeta-F) subscales of meta-emotional knowledge, suggesting that the CI and TH groups have comparable meta-emotional abilities in these areas. However, significant differences were noted in the competence subscale (AvMeta-C) of meta-emotional self-evaluation, with the CI group showing higher scores, indicating a notable overestimation of their abilities in emotional understanding tasks.

Furthermore, the data revealed sex differences within the CI group that were not present in the TH group. Girls with CIs exhibited higher meta-emotional beliefs about using emotions in everyday life compared to boys. Additionally, girls with CIs tended to overestimate their ability to facilitate emotions during the emotional ability test more than boys did. These findings suggest that while both boys and girls with CIs face unique emotional challenges, girls might place more importance on the role of emotions in their daily lives and have higher confidence in their emotional abilities.

### Limitations

Despite the valuable insights provided by this study, several limitations should be acknowledged. First, the cross-sectional design limits the ability to draw causal conclusions about the development of meta-emotional beliefs and self-evaluation over time. Longitudinal studies are needed to track the evolution of these variables and the long-term impact of cochlear implantation on emotional and cognitive development. Second, the sample size, although sufficient for detecting significant differences, may not fully capture the variability within the CI and TH populations. Future studies should include larger and more diverse samples to enhance the generalizability of the findings. Finally, the study focused on a specific age range (10–18 years) and may not account for differences that could emerge in younger children or older adolescents. Expanding the age range in future research could provide a more nuanced view of how meta-emotional intelligence develops across different stages of childhood and adolescence.

### Implications and Future Directions

The findings of this study have several important implications. First, the heightened meta-emotional beliefs and overestimation of emotional abilities in the CI group suggest a need for targeted emotional and meta-emotional skills training for individuals with cochlear implants. Such interventions could help align their self-perceptions with actual abilities, fostering more accurate self-assessment and better emotional regulation. Overestimating one’s emotional abilities may place individuals at greater risk for poor emotional regulation, psychological distress, and social difficulties. This overconfidence can lead to maladaptive coping strategies and peer rejection, potentially increasing susceptibility to anxiety and depression. Recognizing and addressing these issues is crucial and should be considered in mental health and educational interventions for this population. Additionally, the results highlight the importance of considering emotional, meta-emotional, and cognitive factors in the management and support of individuals with cochlear implants. Clinicians and educators should be aware of the potential for overestimation of emotional abilities and work to provide appropriate feedback, training, and support. Future research should explore the underlying mechanisms driving these differences, such as the impact of early auditory deprivation and subsequent auditory rehabilitation on emotional development. Longitudinal studies could provide valuable insights into how these emotional profiles evolve over time and the long-term effects of cochlear implantation on emotional and social functioning.

## Conclusions

In conclusion, this study demonstrates significant differences in meta-emotional intelligence between preadolescents and adolescents with cochlear implants and their typically hearing peers. The heightened meta-emotional beliefs and overestimation of emotional abilities in the CI group underscore the distinct meta-emotional intelligence profile associated with hearing loss and cochlear implantation.

## Data Availability

The data that support the findings of this study are available on request from the corresponding author. The data are not publicly available due to privacy or ethical restrictions.
